# Synthesis of Hydroxytyrosyl Alkyl Ethers from Olive Oil Waste Waters

**DOI:** 10.3390/molecules14051762

**Published:** 2009-05-11

**Authors:** Andrés Madrona, Gema Pereira-Caro, Raquel Mateos, Guillermo Rodríguez, Mariana Trujillo, Juan Fernández-Bolaños, José L. Espartero

**Affiliations:** 1 Dpto. Química Orgánica y Farmacéutica, Facultad de Farmacia, C/ Prof. García González, 2, Universidad de Sevilla, 41012-Sevilla, Spain; 2 Centro Venta del Llano (IFAPA), 23620-Mengíbar (Jaén), Spain; E-mail: mariar.mateos@juntadeandalucia.es (R.M.); 3 Instituto de la Grasa (CSIC), Avda. Padre García Tejero, 4, 41012-Sevilla, Spain; E-mail: jfbg@cica.es (J.F-B.)

**Keywords:** Ethers, hydroxytyrosol, natural products, antioxidants, olive oil, waste waters

## Abstract

The preparation of a new type of derivatives of the naturally occurring antioxidant hydroxytyrosol is reported. Hydroxytyrosyl alkyl ethers were obtained in high yield by a three-step procedure starting from hydroxytyrosol isolated from olive oil waste waters. Preliminary results obtained by the Rancimat method have shown that these derivatives retain the high protective capacity of free hydroxytyrosol.

## 1. Introduction

In the production of Virgin Olive Oil (VOO) 80% of the olive fruit is discarded [1]. In this way, over 10 million tons per year of solid or semisolid wastes are produced worldwide in the olive industry, whose storage and/or recycling represent a serious environmental problem due to its high content in organic matter [2]. These wastes are rich in polyphenols, including hydroxytyrosol (**1**), which are scarcely soluble in the VOO extracted in the process [3]. On the other hand, the small fraction of liposoluble hydroxytyrosol derivatives passing into the VOO is sufficient to protect it from oxidation and rancidity throughout their lifespan [4]. In fact, it has been shown that hydroxytyrosol is superior in protecting fatty foods against oxidation than other antioxidants used in food such as butylated hydroxytoluene (BHT) or α–tocopherol [5]. So, in an effort to exploit this natural residue, several procedures for the isolation and purification of hydroxytyrosol from olive industry wastes have been reported [6,7,8,9,10,11,12,13]. Simultaneously, an explosion in research into the biological properties of hydroxytyrosol has occurred during the past few years, and, as a consequence, it has been demonstrated that this biophenol presents diverse interesting activities, including antimicrobial, hypotensive, hypoglycemic, platelet anti-aggregation, cardioprotective, and anti-inflammatory activities, and also inhibition of several lipoxygenases, and apoptosis induction in HL-60 cells, among others [14,15,16,17,18]. Moreover, an effort in the synthesis of hydroxytyrosyl derivatives with a better hydrophile/lipophile balance (HLB) has been carried out, for their possible use in the protection of fatty foods against oxidation, as well as to increase its bioavailability in the body. In this sense, the syntheses of isochromans [19,20,21] and esters [22,23,24,25,26,27] derivatives have been published in recent years. Such derivatives have shown similar or even improved activities [28] with respect to hydroxytyrosol itself, proving to be, in most cases, more liposolubles. In this short communication, the synthesis of a new type of hydroxytyrosol derivative is presented: alkyl hydroxytyrosyl ethers (**4a**–**h**).

## 2. Results and Discussion

**Scheme 1:**
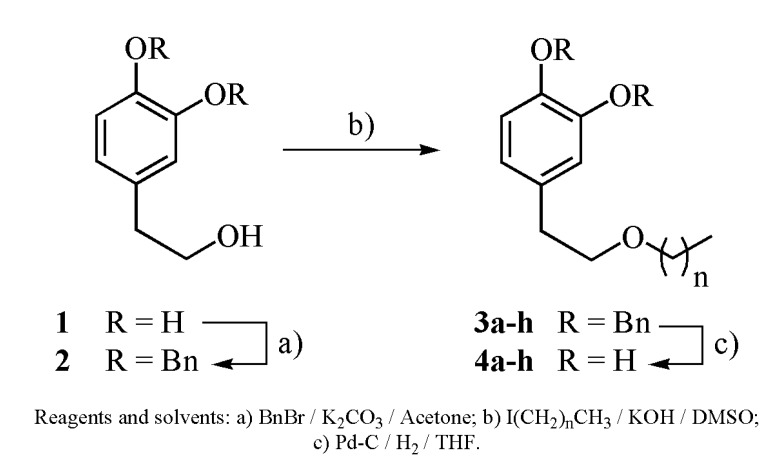
Synthesis of alkyl hydroxytyrosyl ethers.

For their syntheses, hydroxytyrosol isolated from olive oil waste waters (OOWW) was used in order to give an added value to this type of residue. Pure hydroxytyrosol (**1**) was transformed into its known dibenzyl derivative (**2**) [29] by reaction with benzyl bromide/potassium carbonate in acetone. Alkylation of the free alcoholic group with the corresponding alkyl iodides yielded the intermediate compounds **3a**–**h**, in good to excellent yields. The desired alkyl hydroxytyrosyl ethers **4a**–**h** were obtained in excellent yields after hydrogenolytic cleavage of the protecting Bn groups (Scheme 1). The results are summarized in Table 1.

**Table 1:** Yields (%) of pure compounds obtained.

entry	n	**Alkylation Product *(R = Bn)***	Yield (%)	**Deprotection Product *(R = H)***	Yield (%)
1	0	**3a**	91	**4a**	96
2	1	**3b**	86	**4b**	88
3	2	**3c**	78	**4c**	91
4	3	**3d**	84	**4d**	98
5	5	**3e**	82	**4e**	91
6	7	**3f**	80	**4f**	83
7	11	**3g**	67	**4g**	82
8	17	**3h**	60	**4h**	98

New compounds (**3a**–**h** and **4a**–**h**) were characterized by their elemental analyses, and their structures were determined by spectroscopic (NMR and MS) methods. As it can be seen in Table 1, the alkylation step yields decreased as the length of the alkyl chain increased, as a result of the worse solubility of the corresponding alkyl iodide in the media. In fact, in the cases of using *n*-dodecyl (entry 7) and *n*-octadecyl iodide (entry 8), reactions were conducted at 50°C instead of room temperature.

**Figure 1:**
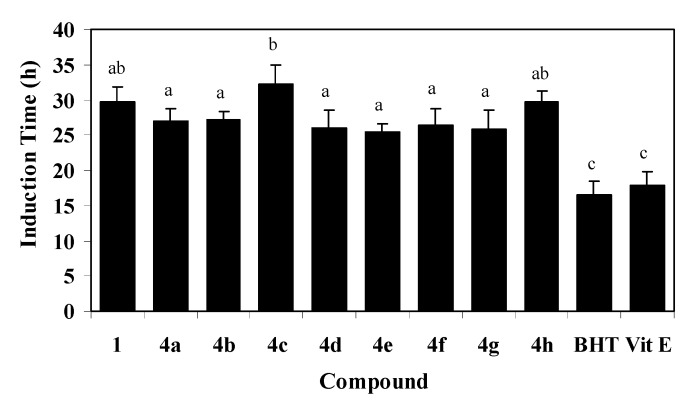
Oxidative stability of lipid matrices spiked with 0,5 mM of hydroxytyrosyl ethers (**4a**–**h**), hydroxytyrosol (**1**), BHT and α–tocopherol (Vit E). Each value is the mean of duplicate measurements ± standard deviation. Results are expressed as Induction Time (IT) in hours. Data with a different letter are statistically different (p < 0.05).

Results on the antioxidant activity of **4a**–**h** have shown that these derivatives maintain the high protective capacity of free hydroxytyrosol [30]. In this way, selected results obtained by the Rancimat^®^ method are shown in Figure 1. As it can be seen, all new compounds **4a**–**h** show similar induction time (IT) values than hydroxytyrosol (**1**) and significantly higher than both BHT and α–tocopherol.

## 3. Experimental

### 3.1. General

All solvent and reagents were of analytical grade unless stated otherwise. Benzyl bromide, palladium over charcoal (Pd/C), and the alkyl iodides (methyl, ethyl, *n*-propyl, *n*-buthyl, *n*-hexyl, *n*-octyl, *n*-dodecyl and *n*-octadecyl iodides) were from Sigma-Aldrich (Steinheim, Germany), as well as 2,6-di-tert-butyl-4-metylphenol (BHT) and α-tocopherol. NMR spectra were recorded on a Bruker Avance 500 spectrophotometer operating at 500.13 MHz (^1^H) and 125.75 MHz (^13^C). Chemical shifts are given in ppm with the residual solvent signals (2.49 ppm for ^1^H and 39.5 ppm for ^13^C) as references. Samples were dissolved (10 mg/mL) in hexadeuterated methylsulfoxide (DMSO-*d_6_*), and spectra were recorded at 303 K. Elemental analyses were made on a Leco CHNS-932 apparatus. High-resolution EI, CI and FAB mass spectra were obtained on a Micromass AUTOSPECQ spectrometer.

### 3.2. Isolation and purification of hydroxytyrosol from olive oil waste waters (OOWW)

OOWW samples were supplied by the ‘Oleícola El Tejar’ oil extraction factory in Córdoba, Spain. These waste samples were partially de-stoned, partially de-oiled (after secondary centrifugal processing to obtain the residual olive oil), and had a high water content (70% of moisture). Thermal treatment between 140 and 180°C, using an operating pressure of 6–10 Kg/cm^2^, for 0.5–1.5 hour was performed in a new semi industrial reactor to allow the maximum phenolic solubilization. Under these conditions a high hydroxytyrosol concentration of up to 2–4 g/L was found in the filtered aqueous phase. After the natural phenolic antioxidant was purified by a patented industrial system in the pilot plant of the Instituto de la Grasa (CSIC, Seville), giving a hydroxytyrosol purity of at least 94% [10]. Further purification by column chromatography using mixtures (1:1 and 2:1) of ethyl ether/hexane as eluants yielded pure hydroxytyrosol (**1**).

### 3.3. Synthetic procedures

*2-(3,4-bis(benzyloxy)phenyl)ethanol* (**2**): To a solution of pure **1** (0.8 g, 5,2 mmol) in dry acetone (25 mL), benzyl bromide (1.4 mL, 11.8 mmol) and potassium carbonate (2.9 g, 20.8 mmol) were added and the resulting mixture heated to reflux for 24 h. The obtained suspension was filtered and concentrated to yield a crude residue, which was further purified by column chromatography, using a 1:2 mixture of diethyl ether/hexane as eluent. The desired product **2** was obtained as a white solid (1.16 g, 67%). M.p.: 55-57°C. All spectroscopic data were in good accordance with those previously reported [29].

#### 3.3.1. General procedure for alkylation of hydroxytyrosol

A mixture of **2** (334 mg, 1 mmol), KOH (335 mg) and the corresponding alkyl iodide (3 mmol) in methyl sulfoxide (12 mL) was stirred at room temperature until completion of reaction (TLC). 3M HCl (25 mL) was added and the mixture extracted with CHCl_3_ (3 ° 25 mL). The organic phase was washed with 2% NaHSO_3_ (25 mL) and water (25 mL), dried over Na_2_SO_4_, filtered and evaporated. The desired products **3a**–**h** were purified by flash column chromatography over silica gel.

*1,2-bis(Benzyloxy)-4-(2-methoxyethyl)benzene* (**3a**): colourless liquid (91% yield); ^1^H-NMR δ ppm 7.37 (m, 10H, 2 x *Ph*), 6.95 (d, *J* = 2.0 Hz, 1H, *H_4_*), 6.93 (d, *J* = 8.2 Hz, 1H, *H_7_*), 6.72 (dd, *J* = 2.0 Hz, *J* = 8.2 Hz, 1H, *H_8_*), 5.09 (s, 2H, C*H_2_*Ph in pos. 5), 5.07 (s, 2H, C*H_2_*Ph in pos. 6), 3.46 (t, *J* = 7.0 Hz, 2H, *H_1_*), 3.21 (s, 3H, *H_1’_*), 2.69 (t, *J* = 7.0 Hz, 2H, *H_2_*); ^13^C-NMR δ ppm 148.1 (*C_5_*), 146.6 (*C_6_*), 137.5 and 137.4 (*C_ipso_, Bn groups*), 132.1 (*C_3_*), 128.3-127.4 (*C_3_, C_4_ and C_5_, Bn groups*), 121.3 (*C_8_*), 115.4 (*C_4_*), 114.7 (*C_7_*), 72.9 (*C_1_*), 70.3 (*C*H_2_Ph in pos. 6), 70.1 (*C*H_2_Ph in pos. 5), 57.7 (*C_1’_*), 34.8 (*C_2_*); Elem. anal. calc. for C_23_H_24_O_3_ C, 79.28; H, 6.94; found: C, 78.75; H, 6.87; HRMS, 348.172247 (0.9 ppm).

*1,2-bis(Benzyloxy)-4-(2-ethoxyethyl)benzene* (**3b**): colourless liquid (86% yield); ^1^H-NMR δ ppm 7.37 (m, 10H, 2 x *Ph*), 6.96 (d, *J* = 2.0 Hz, 1H, *H_4_*), 6.93 (d, *J* = 8.2 Hz, 1H, *H_7_*), 6.72 (dd, *J* = 2.0 Hz, *J* = 8.2 Hz, 1H, *H_8_*), 5.09 (s, 2H, C*H_2_*Ph in pos. 5), 5.07 (s, 2H, C*H_2_*Ph in pos. 6), 3.49 (t, *J* = 7.0 Hz, 2H, *H_1_*), 3.39 (q, *J* = 7.0 Hz, 2H, *H_1’_*), 2.69 (t, *J* = 7.0 Hz, 2H, *H_2_*), 1.07 (t, *J* = 7.0 Hz, 3H, *H_2’_*); ^13^C- NMR δ ppm 148.1 (*C_5_*), 146.6 (*C_6_*), 137.5 and 137.4 (*C_ipso_, Bn groups*), 132.2 (*C_3_*), 128.3-127.4 (*C_3_, C_4_ and C_5_, Bn groups*), 121.3 (*C_8_*), 115.4 (*C_4_*), 114.7 (*C_7_*), 70.7 (*C_1_*), 70.2 (*C*H_2_Ph in pos. 6), 70.1 (*C*H_2_Ph in pos. 5), 65.1 (*C_1’_*), 35.1 (*C_2_*), 15.0 (*C_2’_*); Elem. anal. calc. for C_24_H_26_O_3_ x ½ H_2_O C, 77.60; H, 7.33; found: C, 77.99; H, 6.91; HRMS, 362.189060 (2.4 ppm).

*1,2-bis(Benzyloxy)-4-(2-propoxyethyl)benzene* (**3c**): colourless liquid (78% yield); ^1^H-NMR δ ppm 7.37 (m, 10H, 2 x *Ph*), 6.96 (d, *J* = 2.0 Hz, 1H, *H_4_*), 6.93 (d, *J* = 8.2 Hz, 1H, *H_7_*), 6.72 (dd, *J* = 2.0 Hz, *J* = 8.2 Hz, 1H, *H_8_*), 5.09 (s, 2H, C*H_2_*Ph in pos. 5), 5.07 (s, 2H, C*H_2_*Ph in pos. 6), 3.49 (t, *J* = 7.1 Hz, 2H, *H_1_*), 3.30 (t, *J* = 6.6 Hz, 2H, *H_1’_*), 2.69 (t, *J* = 7.1 Hz, 2H, *H_2_*), 1.47 (m, 2H, *H_2’_*), 0.83 (t, *J* = 7.4 Hz, 3H, *H_3’_*); ^13^C-NMR δ ppm 148.1 (*C_5_*), 146.6 (*C_6_*), 137.4 and 137.3 (*C_ipso_, Bn groups*), 132.2 (*C_3_*), 128.3-127.4 (*C_3_, C_4_ and C_5_, Bn groups*), 121.3 (*C_8_*), 115.5 (*C_4_*), 114.7 (*C_7_*), 71.5 (*C_1’_*), 70.9 (*C_1_*), 70.2 (*C*H_2_Ph in pos. 6), 70.1 (*C*H_2_Ph in pos. 5), 35.1 (*C_2_*), 22.4 (*C_2’_*) , 10.4 (*C_3’_*); Elem. anal. calc. for C_25_H_28_O_3_ C, 79.75; H, 7.50; found: C, 79.15; H, 6.88; HRMS, 376.205160 (3.5 ppm).

*1,2-bis(Benzyloxy)-4-(2-butoxyethyl)benzene* (**3d**): colourless liquid (84% yield); ^1^H-NMR δ ppm 7.37 (m, 10H, 2 x *Ph*), 6.96 (d, *J* = 2.0 Hz, 1H, *H_4_*), 6.93 (d, *J* = 8.2 Hz, 1H, *H_7_*), 6.72 (dd, *J* = 2.0 Hz, *J* = 8.2 Hz, 1H, *H_8_*), 5.08 (s, 2H, C*H_2_*Ph in pos. 5), 5.07 (s, 2H, C*H_2_*Ph in pos. 6), 3.49 (t, *J* = 7.0 Hz, 2H, *H_1_*), 3.34 (t, *J* = 6.5 Hz, 2H, *H_1’_*), 2.69 (t, *J* = 7.0 Hz, 2H, *H_2_*), 1.44 (m, 2H, *H_2’_*), 1.28 (m, 2H, *H_3’_*), 0.85 (t, *J* = 7.4 Hz, 3H, *H_4’_*); ^13^C-NMR δ ppm 148.1 (*C_5_*), 146.6 (*C_6_*), 137.5 and 137.4 (*C_ipso_, Bn groups*), 132.2 (*C_3_*), 128.3-127.4 (*C_3_, C_4_ and C_5_, Bn groups*), 121.3 (*C_8_*), 115.5 (*C_4_*), 114.7 (*C_7_*), 71.0 (*C_1_*), 70.2 (*C*H_2_Ph in pos. 6), 70.1 (*C*H_2_Ph in pos. 5), 69.6 (*C_1’_*), 35.1 (*C_2_*), 31.3 (*C_2’_*), 18.8 (*C_3’_*), 13.7 (*C_4’_*); Elem. anal. calc. for C_26_H_30_O_3_ C, 79.97; H, 7.74; found: C, 79.52; H, 7.38; HRMS, 390.218471 (2.6 ppm).

*1,2-bis(Benzyloxy)-4-(2-(hexyloxy)ethyl)benzene* (**3e**): colourless liquid (82% yield); ^1^H-NMR δ ppm 7.37 (m, 10H, 2 x *Ph*), 6.96 (d, *J* = 2.0 Hz, 1H, *H_4_*), 6.93 (d, *J* = 8.2 Hz, 1H, *H_7_*), 6.72 (dd, *J* = 2.0 Hz, *J* = 8.2 Hz, 1H, *H_8_*), 5.08 (s, 2H, C*H_2_*Ph in pos. 5), 5.07 (s, 2H, C*H_2_*Ph in pos. 6), 3.49 (t, *J* = 7.0 Hz, 2H, *H_1_*), 3.33 (t, *J* = 6.5 Hz, 2H, *H_1’_*), 2.69 (t, *J* = 7.0 Hz, 2H, *H_2_*), 1.45 (m, 2H, *H_2’_*), 1.24 (m, 6H, *H_3’_*−*H_5’_*), 0.84 (t, *J* = 7.0 Hz, 3H, *H_6’_*); ^13^C-NMR δ ppm 148.1 (*C_5_*), 146.6 (*C_6_*), 137.5 and 137.4 (*C_ipso_, Bn groups*), 132.2 (*C_3_*), 128.3-127.4 (*C_3_, C_4_ and C_5_, Bn groups*), 121.3 (*C_8_*), 115.5 (*C_4_*), 114.7 (*C_7_*), 71.0 (*C_1_*), 70.2 (*C*H_2_Ph in pos. 6), 70.1 (*C*H_2_Ph in pos. 5), 69.9 (*C_1’_*), 35.1 (*C_2_*), 31.0 (*C_4’_*), 29.1 (*C_2’_*), 25.3 (*C_3’_*), 22.0 (*C_5’_*), 13.8 (*C_6’_*); Elem. anal. calc. for C_28_H_34_O_3_ C, 80.35; H, 8.19; found: C, 79.55; H, 7.86; HRMS, 418.250377 (1.0 ppm).

*1,2-bis(Benzyloxy)-4-(2-(octyloxy)ethyl)benzene* (**3f**): white solid (80% yield); mp 52−54°C; ^1^H- NMR δ ppm 7.37 (m, 10H, 2 x *Ph*), 6.96 (d, *J* = 2.0 Hz, 1H, *H_4_*), 6.93 (d, *J* = 8.2 Hz, 1H, *H_7_*), 6.72 (dd, *J* = 2.0 Hz, *J* = 8.2 Hz, 1H, *H_8_*), 5.08 (s, 2H, C*H_2_*Ph in pos. 5), 5.07 (s, 2H, C*H_2_*Ph in pos. 6), 3.49 (t, *J* = 7.0 Hz, 2H, *H_1_*), 3.33 (t, *J* = 6.5 Hz, 2H, *H_1’_*), 2.69 (t, *J* = 7.0 Hz, 2H, *H_2_*), 1.45 (m, 2H, *H_2’_*), 1.23 (m, 10H, *H_3’_*−*H_7’_*), 0.84 (t, *J* = 7.0 Hz, 3H, *H_8’_*); ^13^C-NMR δ ppm 148.1 (*C_5_*), 146.6 (*C_6_*), 137.4 and 137.3 (*C_ipso_, Bn groups*), 132.2 (*C_3_*), 128.2-127.4 (*C_3_, C_4_ and C_5_, Bn groups*), 121.3 (*C_8_*), 115.4 (*C_4_*), 114.7 (*C_7_*), 71.0 (*C_1_*), 70.2 (*C*H_2_Ph in pos. 6), 70.1 (*C*H_2_Ph in pos. 5), 69.9 (*C_1’_*), 35.1 (*C_2_*), 31.2 (*C_6’_*), 29.1 (*C_2’_*), 28.7 (*C_4’_*), 28.6 (*C_5’_*), 25.7 (*C_3’_*), 22.0 (*C_7’_*), 13.8 (*C_8’_*); Elem. anal. calc. for C_30_H_38_O_3_ C, 80.68; H, 8.58; found: C, 79.93; H, 8.75; HRMS, 446.282509 (0.9 ppm).

*1,2-bis(Benzyloxy)-4-(2-(dodecyloxy)ethyl)benzene* (**3g**): white solid (67% yield); mp 42−45°C; ^1^H- NMR δ ppm 7.37 (m, 10H, 2 x *Ph*), 6.96 (d, *J* = 2.0 Hz, 1H, *H_4_*), 6.93 (d, *J* = 8.2 Hz, 1H, *H_7_*), 6.72 (dd, *J* = 2.0 Hz, *J* = 8.2 Hz, 1H, *H_8_*), 5.08 (s, 2H, C*H_2_*Ph in pos. 5), 5.06 (s, 2H, C*H_2_*Ph in pos. 6), 3.49 (t, *J* = 7.0 Hz, 2H, *H_1_*), 3.33 (t, *J* = 6.5 Hz, 2H, *H_1’_*), 2.69 (t, *J* = 7.0 Hz, 2H, *H_2_*), 1.45 (m, 2H, *H_2’_*), 1.22 (m, 18H, *H_3’_*−*H_11’_*), 0.84 (t, *J* = 7.0 Hz, 3H, *H_12’_*); ^13^C-NMR δ ppm 148.1 (*C_5_*), 146.6 (*C_6_*), 137.4 and 137.3 (*C_ipso_, Bn groups*), 132.2 (*C_3_*), 128.2-127.4 (*C_3_, C_4_ and C_5_, Bn groups*), 121.3 (*C_8_*), 115.4 (*C_4_*), 114.7 (*C_7_*), 70.9 (*C_1_*), 70.3 (*C*H_2_Ph in pos. 6), 70.1 (*C*H_2_Ph in pos. 5), 69.9 (*C_1’_*), 35.1 (*C_2_*), 31.2 (*C_10’_*), 29.1 (*C_2’_*), 29.0−28.6 (*C_4’_*−*C_9’_*), 25.7 (*C_3’_*), 22.0 (*C_11’_*), 13.8 (*C_12’_*); Elem. anal. calc. for C_34_H_46_O_3_ C, 81.23; H, 9.22; found: C, 81.01; H, 9.07; HRMS, 502.344735 (0.1 ppm).

*1,2-bis(Benzyloxy)-4-(2-(octadecyloxy)ethyl)benzene* (**3h**): white solid (60% yield); mp 57−59°C; ^1^H-NMR δ ppm 7.37 (m, 10H, 2 x *Ph*), 6.96 (d, *J* = 2.0 Hz, 1H, *H_4_*), 6.93 (d, *J* = 8.2 Hz, 1H, *H_7_*), 6.72 (dd, *J* = 2.0 Hz, *J* = 8.2 Hz, 1H, *H_8_*), 5.08 (s, 2H, C*H_2_*Ph in pos. 5), 5.06 (s, 2H, C*H_2_*Ph in pos. 6), 3.49 (t, *J* = 7.0 Hz, 2H, *H_1_*), 3.33 (t, *J* = 6.5 Hz, 2H, *H_1’_*), 2.69 (t, *J* = 7.0 Hz, 2H, *H_2_*), 1.45 (m, 2H, *H_2’_*), 1.22 (m, 30H, *H_3’_*−*H_17’_*), 0.84 (t, *J* = 7.0 Hz, 3H, *H_18’_*); ^13^C-NMR δ ppm 148.1 (*C_5_*), 146.6 (*C_6_*), 137.4 and 137.3 (*C_ipso_, Bn groups*), 132.2 (*C_3_*), 128.2-127.4 (*C_3_, C_4_ and C_5_, Bn groups*), 121.3 (*C_8_*), 115.5 (*C_4_*), 114.7 (*C_7_*), 70.9 (*C_1_*), 70.2 (*C*H_2_Ph in pos. 6), 70.1 (*C*H_2_Ph in pos. 5), 69.8 (*C_1’_*), 35.0 (*C_2_*), 31.2 (*C_16’_*), 29.1 (*C_2’_*), 29.0−28.6 (*C_4’_*−*C_15’_*), 25.6 (*C_3’_*), 22.0 (*C_17’_*), 13.8 (*C_18’_*); Elem. anal. calc. for C_40_H_58_O_3_ C, 81.86; H, 9.96; found: C, 81.77; H, 10.00; HRMS, 586.438530 (0.1 ppm).

#### 3.3.2. General procedure for cleavage of Bn protective groups

Palladium over charcoal (Pd-C) was added to a solution of the corresponding ether (**3a**–**h**, 1 mmol) in THF (20 mL) and the mixture was hydrogenated at 4 bar with magnetic stirring. After 24 h at room temperature the catalyst was filtered off and solvent was evaporated in vacuum, yielding the desired compound in each case (**4a**–**h**) that was purified by column chromatography.

*4-(2-Methoxyethyl)benzene-1,2-diol* (**4a**): colourless liquid (96% yield); ^1^H-NMR δ ppm 6.60 (d, *J* = 8.0 Hz, 1H, *H_7_*), 6.58 (d, *J* = 2.1 Hz, 1H, *H_4_*), 6.43 (dd, *J* = 2.1 Hz, *J* = 8.0 Hz, 1H, *H_8_*), 3.42 (t, *J* = 7.0 Hz, 2H, *H_1_*), 3.21 (s, 3H, *H_1’_*), 2.60 (t, *J* = 7.0 Hz, 2H, *H_2_*); ^13^C-NMR δ ppm 144.9 (*C_5_*), 143.4 (*C_6_*), 129.7 (*C_3_*), 119.3 (*C_8_*), 116.2 (*C_4_*), 115.3 (*C_7_*), 73.2 (*C_1_*), 57.7 (*C_1’_*), 35.0 (*C_2_*); Elem. anal. calc. for C_9_H_12_O_3_ C, 64.27; H, 7.19; found: C, 63.74; H, 6.94; HRMS, 168.079247 (3.6 ppm).

*4-(2-Ethoxyethyl)benzene-1,2-diol* (**4b**): colourless liquid (88% yield); ^1^H-NMR δ ppm 6.60 (d, *J* = 8.0 Hz, 1H, *H_7_*), 6.58 (d, *J* = 2.1 Hz, 1H, *H_4_*), 6.44 (dd, *J* = 2.1 Hz, *J* = 8.0 Hz, 1H, *H_8_*), 3.45 (t, *J* = 7.2 Hz, 2H, *H_1_*), 3.40 (q, *J* = 7.0 Hz, 2H, *H_1’_*), 2.59 (t, *J* = 7.2 Hz, 2H, *H_2_*), 1.08 (t, *J* = 7.0 Hz, 3H, *H_2’_*); ^13^C- NMR δ ppm 144.9 (*C_5_*), 143.2 (*C_6_*), 129.7 (*C_3_*), 119.3 (*C_8_*), 116.2 (*C_4_*), 115.3 (*C_7_*), 71.1 (*C_1_*), 65.1 (*C_1’_*), 35.0 (*C_2_*), 15.0 (*C_2’_*); Elem. anal. calc. for C_10_H_14_O_3_ C, 65.91; H, 7.74; found: C, 65.39; H, 7.44; HRMS, 182.094382 (0.5 ppm).

*4-(2-Propoxyethyl)benzene-1,2-diol* (**4c**): white solid (91% yield); mp 93−95°C; ^1^H-NMR δ ppm 6.60 (d, *J* = 8.0 Hz, 1H, *H_7_*), 6.58 (d, *J* = 2.1 Hz, 1H, *H_4_*), 6.44 (dd, *J* = 2.1 Hz, *J* = 8.0 Hz, 1H, *H_8_*), 3.45 (t, *J* = 7.2 Hz, 2H, *H_1_*), 3.31 (t, *J* = 6.6 Hz, 2H, *H_1’_*), 2.60 (t, *J* = 7.2 Hz, 2H, *H_2_*), 1.48 (m, 2H, *H_2’_*), 0.83 (t, *J* = 7.4 Hz, 3H, *H_3’_*); ^13^C-NMR δ ppm 144.9 (*C_5_*), 143.3 (*C_6_*), 129.7 (*C_3_*), 119.3 (*C_8_*), 116.2 (*C_4_*), 115.3 (*C_7_*), 71.5 (*C_1’_*), 71.3 (*C_1_*), 35.0 (*C_2_*), 22.4 (*C_2’_*), 10.5 (*C_3’_*); Elem. anal. calc. for C_11_H_16_O_3_ x ⅓ H_2_O C, 65.32; H, 8.31; found: C, 65.29; H, 7.76; HRMS, 196.109392 (2.8 ppm).

*4-(2-Butoxyethyl)benzene-1,2-diol* (**4d**): white solid (98% of yield); mp 66−68°C; ^1^H-NMR δ ppm 6.60 (d, *J* = 8.0 Hz, 1H, *H_7_*), 6.58 (d, *J* = 2.1 Hz, 1H, *H_4_*), 6.44 (dd, *J* = 2.1 Hz, *J* = 8.0 Hz, 1H, *H_8_*), 3.45 (t, *J* = 7.2 Hz, 2H, *H_1_*), 3.34 (t, *J* = 6.6 Hz, 2H, *H_1’_*), 2.59 (t, *J* = 7.2 Hz, 2H, *H_2_*), 1.45 (m, 2H, *H_2’_*), 1.29 (m, 2H, *H_3’_*), 0.85 (t, *J* = 7.4 Hz, 3H, *H_4’_*); ^13^C-NMR δ ppm 144.8 (*C_5_*), 143.3 (*C_6_*), 129.7 (*C_3_*), 119.3 (*C_8_*), 116.2 (*C_4_*), 115.3 (*C_7_*), 71.4 (*C_1_*), 69.6 (*C_1’_*), 35.0 (*C_2_*), 31.3 (*C_2’_*), 18.8 (*C_3’_*), 13.7 (*C_4’_*); Elem. anal. calc. for C_12_H_18_O_3_ C, 68.54; H, 8.63; found: C, 68.09; H, 8.54; HRMS, 210.125280 (1.5 ppm).

*4-(2-(Hexyloxy)ethyl)benzene-1,2-diol* (**4e**): colourless liquid (91% yield); ^1^H-NMR δ ppm 6.60 (d, *J* = 8.0 Hz, 1H, *H_7_*), 6.58 (d, *J* = 2.1 Hz, 1H, *H_4_*), 6.44 (dd, *J* = 2.1 Hz, *J* = 8.0 Hz, 1H, *H_8_*), 3.45 (t, *J* = 7.2 Hz, 2H, *H_1_*), 3.34 (t, *J* = 6.6 Hz, 2H, *H_1’_*), 2.59 (t, *J* = 7.2 Hz, 2H, *H_2_*), 1.45 (m, 2H, *H_2’_*), 1.24 (m, 6H, *H_3’_*−*H_5’_*), 0.85 (t, *J* = 7.1 Hz, 3H, *H_6’_*); ^13^C-NMR δ ppm 144.9 (*C_5_*), 143.3 (*C_6_*), 129.7 (*C_3_*), 119.3 (*C_8_*), 116.2 (*C_4_*), 115.3 (*C_7_*), 71.4 (*C_1_*), 69.9 (*C_1’_*), 35.0 (*C_2_*), 31.0 (*C_4’_*), 29.1 (*C_2’_*), 25.3 (*C_3’_*), 22.0 (*C_5’_*), 13.8 (*C_6’_*); Elem. anal. calc. for C_14_H_22_O_3_ x ⅓ H_2_O C, 68.82; H, 9.35; found: C, 69.22; H, 8.88; HRMS, 238.157767 (3.7 ppm).

*4-(2-(Octyloxy)ethyl)benzene-1,2-diol* (**4f**): colourless liquid (83% yield); ^1^H-NMR δ ppm 6.60 (d, *J* = 8.0 Hz, 1H, *H_7_*), 6.58 (d, *J* = 2.1 Hz, 1H, *H_4_*), 6.43 (dd, *J* = 2.1 Hz, *J* = 8.0 Hz, 1H, *H_8_*), 3.44 (t, *J* = 7.2 Hz, 2H, *H_1_*), 3.33 (t, *J* = 6.6 Hz, 2H, *H_1’_*), 2.59 (t, *J* = 7.2 Hz, 2H, *H_2_*), 1.45 (m, 2H, *H_2’_*), 1.23 (m, 10H, *H_3’_*−*H_7’_*), 0.85 (t, *J* = 7.0 Hz, 3H, *H_8’_*); ^13^C-NMR δ ppm 144.8 (*C_5_*), 143.3 (*C_6_*), 129.7 (*C_3_*), 119.3 (*C_8_*), 116.1 (*C_4_*), 115.3 (*C_7_*), 71.4 (*C_1_*), 69.9 (*C_1’_*), 35.0 (*C_2_*), 31.2 (*C_6’_*), 29.1 (*C_2’_*), 28.7 (*C_4’_*), 28.6 (*C_5’_*), 25.6 (*C_3’_*), 22.0 (*C_7’_*), 13.8 (*C_8’_*); HRMS, 266.188001 (0.7 ppm).

*4-(2-(Dodecyloxy)ethyl)benzene-1,2-diol* (**4g**): white solid (82% yield); mp 39−41°C; ^1^H-NMR δ ppm 6.60 (d, *J* = 8.0 Hz, 1H, *H_7_*), 6.57 (d, *J* = 2.1 Hz, 1H, *H_4_*), 6.43 (dd, *J* = 2.1 Hz, *J* = 8.0 Hz, 1H, *H_8_*), 3.44 (t, *J* = 7.2 Hz, 2H, *H_1_*), 3.33 (t, *J* = 6.6 Hz, 2H, *H_1’_*), 2.59 (t, *J* = 7.2 Hz, 2H, *H_2_*), 1.45 (m, 2H, *H_2’_*), 1.23 (m, 18H, *H_3’_*−*H_11’_*), 0.84 (t, *J* = 7.0 Hz, 3H, *H_12’_*); ^13^C-NMR δ ppm 144.8 (*C_5_*), 143.3 (*C_6_*), 129.7 (*C_3_*), 119.3 (*C_8_*), 116.1 (*C_4_*), 115.3 (*C_7_*), 71.4 (*C_1_*), 69.9 (*C_1’_*), 35.0 (*C_2_*), 31.2 (*C_10’_*), 29.1 (*C_2’_*), 28.9−28.6 (*C_4’_*−*C_9’_*), 25.6 (*C_3’_*), 22.0 (*C_11’_*), 13.8 (*C_12’_*); Elem. anal. calc. for C_20_H_34_O_3_ C, 74.49; H, 10.63; found: C, 74.11; H, 10.64; HRMS, 322.250251 (1.7 ppm).

*4-(2-(Octadecyloxy)ethyl)benzene-1,2-diol* (**4h**): white solid (98% yield); mp 65−67°C; ^1^H-NMR δ ppm 6.60 (d, *J* = 8.0 Hz, 1H, *H_7_*), 6.57 (d, *J* = 2.1 Hz, 1H, *H_4_*), 6.43 (dd, *J* = 2.1 Hz, *J* = 8.0 Hz, 1H, *H_8_*), 3.44 (t, *J* = 7.2 Hz, 2H, *H_1_*), 3.33 (t, *J* = 6.6 Hz, 2H, *H_1’_*), 2.59 (t, *J* = 7.2 Hz, 2H, *H_2_*), 1.45 (m, 2H, *H_2’_*), 1.23 (m, 30H, *H_3’_*−*H_17’_*), 0.84 (t, *J* = 7.0 Hz, 3H, *H_18’_*); ^13^C-NMR δ ppm 144.9 (*C_5_*), 143.3 (*C_6_*), 129.7 (*C_3_*), 119.3 (*C_8_*), 116.1 (*C_4_*), 115.3 (*C_7_*), 71.4 (*C_1_*), 69.9 (*C_1’_*), 35.0 (*C_2_*), 31.2 (*C_16’_*), 29.2 (*C_2’_*), 29.0−28.6 (*C_4’_*−*C_15’_*), 25.6 (*C_3’_*), 22.0 (*C_17’_*), 13.8 (*C_18’_*); Elem. anal. calc. for C_26_H_46_O_3_ x ⅓ H_2_O C, 75.68; H, 11.40; found: C, 75.77; H, 12.20; HRMS, 406.344308 (1.0 ppm).

### 3.4. Evaluation of oxidative stability of lipid matrices

The oxidative stability of a lipid matrix, obtained from commercial sunflower oil, was evaluated by an automated test using the Rancimat apparatus (Model 743, Metrohm Co. Basel, Switzerland). Aliquots of the purified glyceridic matrix [30] were spiked with 0.5 mM of antioxidant and subjected to accelerated oxidation. A flow of air (15 L/h) was bubbled successively through the matrices and heated at 90°C. In this process, the volatile oxidation products are stripped from the oil and dissolved in the water, increasing the water conductivity. The time taken until there is a sharp increase of conductivity is named induction time (IT) and is expressed in hours. All determinations were carried out by duplicate. Data were subjected to a one-way analysis of variance (ANOVA) using Statistix 8.0. Differences were considered significant when p < 0.05.

## 4. Conclusions

In conclusion, we have designed and easily synthesized new alkyl hydroxytyrosyl ethers (**4a**–**h**) in high yield as potential antioxidant additives. All the new compounds have been completely characterized by spectroscopic methods. Free hydroxytyrosol recovered and purified from olive oil waste waters has been used as starting material. Results obtained by the Rancimat method have shown that these derivatives maintain the high protective capacity of free hydroxytyrosol, and further studies are being carried out to determine the bioavailability and toxicity of these derivatives.
